# Human papillomavirus type 16 E6 induces cell competition

**DOI:** 10.1371/journal.ppat.1010431

**Published:** 2022-03-23

**Authors:** Nicole Brimer, Scott Vande Pol

**Affiliations:** Department of Pathology, University of Virginia, Charlottesville, Virginia, United States of America; Tufts University School of Medicine, UNITED STATES

## Abstract

High-risk human papillomavirus (HPV) infections induce squamous epithelial tumors in which the virus replicates. Initially, the virus-infected cells are untransformed, but expand in both number and area at the expense of uninfected squamous epithelial cells. We have developed an *in vitro* assay in which colonies of post-confluent HPV16 expressing cells outcompete and displace confluent surrounding uninfected keratinocytes. The enhanced colony competition induced by the complete HPV16 genome is conferred by E6 expression alone, not by individual expression of E5 or E7, and requires E6 interaction with p53. E6-expressing keratinocytes undermine and displace adjacent normal keratinocytes from contact with the attachment substrate, thereby expanding the area of the E6-expressing colony at the expense of normal keratinocytes. These new results separate classic oncogenicity that is primarily conferred by HPV16 E7 from cell competition that we show is primarily conferred by E6 and provides a new biological role for E6 oncoproteins from high-risk human papillomaviruses.

## Author summary

Microbial infections can change the fate and behavior of normal vertebrate cells to resemble oncogenic cells. High-risk papillomaviruses induce infected squamous epithelial cells to form tumors, some of which evolve into malignancies. The present work shows that the enhanced competitiveness of HPV16-infected cells for the basal cell surface is primarily due to the expression of the E6 oncoprotein and not the E7 or E5 oncoproteins. Compared to normal keratinocytes, E6 induces a super-competitor phenotype while E5 and E7 do not. This work shows the importance of measuring oncoprotein traits not only as cell autonomous traits, but in the context of competition with uninfected cells and shows the potential of papillomavirus oncoproteins to be novel genetic probes for the analysis of cell competition.

## Introduction

Papillomaviruses induce epithelial papillomas that can vary in size from visually inapparent up to kilogram masses [1]. The virus replicates within the papilloma under the control of virus-encoded E1 and E2 proteins [2,3]. The virally-encoded E5, E6, and E7 oncoproteins contribute to the formation of the papilloma [4–6], and are expressed under the transcriptional control of cellular transcription factors together with the E1 and E2 proteins [7–14]. In some HPV types and in Bovine Papillomavirus type I, the complete papillomavirus replication cycle can be studied in vitro using keratinocyte organotypic culture and cloned viral DNA [15–19].

The papillomavirus infection cycle begins with exposure of basal epithelial cells and the basement membrane to a virus inoculum; virus associates with the basement membrane, is taken up by basal epithelial cells, and early viral genes including the viral oncoproteins are expressed [20]. The initially infected cell(s) must attach to and persist on the basement membrane, because if the attachment is lost, the infected cell(s) could be forced apically by other more basal proliferating cells, resulting in the loss of the infected cell by desquamation from the epithelial surface. Therefore, attachment to the basement membrane and the ability of daughter cells to remain attached and proliferate at the expense of surrounding uninfected epithelium is a requirement for an incipient papilloma to expand. How virally infected cells outcompete at the expense of uninfected keratinocytes is presumably (but as yet unproven to be) a consequence of viral oncoprotein expression; but which oncoprotein(s) most influences cell competition is as yet unknown.

Cell competition is a rapidly expanding field that originated from the observation that normal cells in *Drosophila* embryos eliminate adjacent cells that are impaired but viable ([21] and references therein). In cell competition, normal cells eliminate other normal cells that have reduced fitness. Described mechanisms include the induction of apoptosis, mechanical competition, and intercellular signaling that induces differentiation (recently reviewed in [22,23]). While the hallmark of cell competition is normal cells eliminating impaired cells, under abnormal conditions, super-competing cells can eliminate normal cells as is seen when super-competing cells produced by overexpression of c-myc can induce the displacement or death of normal surrounding cells [24,25]. Since papillomaviruses induce papillomas that expand at the expense of normal tissues, viral manipulation of cell competition may play a role. The viral oncoproteins E5, E6, and E7 would be candidates to manipulate cell competition. These viral oncoproteins have been characterized by classic oncoprotein assays such as focus formation, anchorage independent colony formation by 3T3 cells, or transgenic expression in murine skin, all of which are all assays that measure the oncoprotein’s traits in homogenous cell populations. Such assays do not recapitulate the early stages of an in vivo infection where a virally infected cell population is observed to progressively expand, successfully enlarging to form a papilloma [26].

In classic assays for oncogene activity, the major oncogene of HPV16 is 16E7, which when compared to 16E6, has increased ability to induce anchorage independent colonies [27,28] and induces a more severe dysplasia than 16E6 when expressed in the skin of mice [29–31]. The E7 oncoproteins are best known for targeting the degradation of Retinoblastoma family proteins [32–34] and the tyrosine phosphatase PTPN14 that is a negative regulator of Hippo signaling [35–37]. If the ability of HPV16 genomes to induce enhanced cell competition were due to classic oncogenic potency, E7 would seem to be the most likely candidate. However, in experiments presented here we find that it is HPV16 E6 and neither E7 nor E5 that independently enhances cell competition.

## Materials and methods

### Cell culture

NIKS Keratinocytes are human foreskin keratinocytes that are both feeder-cell and growth-factor dependent for proliferation, support the complete HPV lifecycle, are untransformed, and have an extended lifespan [38]; they were obtained from ATCC (https://www.atcc.org). NIKS were co-cultured with mitomycin C treated 3T3 cells in F-media and sequentially transduced with replication defective lentiviruses and with replication defective murine retroviruses encoding fluorescent proteins and then viral oncogenes as previously described [39]. NIKS cells were transfected with re-circularized cloned HPV16; episomal status of the HPV16 genome was confirmed by southern blot [40], and then transduced with lentivirus encoding fluorescent proteins, frozen in aliquots and used in assays at early passage. Primary keratinocytes were derived from anonymous discarded neonatal foreskins collected from the University of Virginia Medical Center and classified as non-human subject research, and were maintained and virally transduced in F-media with mitomycin C treated 3T3 cells and Rho Kinase inhibitor (Y-27632, ThermoFisher) as previously described [41]; they were sequentially transduced with lentiviruses and retroviruses as described for NIKS above.

### Plasmids

HPV16 nts 56–879 encompassing the E6 and E7 region cloned into murine retrovirus vector pLXSN was the kind gift of Denise Galloway [42]. Stop codons were introduced at amino acid 12 of E6 and amino acid 8 of E7 either alone or in combination as shown in the figures. HPV16 E5 cloned into a retroviral expression vector was the kind gift of Richard Schlegel (Georgetown University) [43]. EGFP (from Clontech) or Fusion Red [44] (obtained from Addgene clone 54778) were cloned into a lentiviral packaging plasmid with an internal MSCV promoter and puromycin selection.

### Cell competition assay

Primary Keratinocytes cultured in F media in the presence of Y-27632, or NIKS cells, or HPV16 transfected NIKS cells, were cultured in F media with feeder cells as described above. Keratinocytes transduced with either the EGFP or Fusion Red lentivirus were then transduced with the above-described murine retroviruses expressing either wild-type or mutated 16E6, and/or 16E7 or 16E5, and drug-selected in F media with puromycin and G418 (and in the case of primary cells, additionally with 10 uM Y-27632). One day before the beginning of the assay, 99.5% to 99.9% vector-expressing cells and 0.1 to 0.5% oncogene-expressing cells in contrasting fluorescent tagged cells were mixed and plated together at 10% confluency in a 10 cm dish. 24 hours later, those cells were trypsinized and re-plated onto glass coverslips in a 6 well plate (at 2.1 X 10^4^ cells / cm2) together with mitomycin-C treated feeder 3T3 cells in F media (with or without Y27632). Cells typically reached confluency at day 5–7, at which point one well is fixed and a second well is fed with F-media for another 7 days until fixation, and a third well fixed on days 17–21. Coverslips were stained with dapi. Fluorescent images were acquired as 16-bit TIFF images with a Nikon inverted TE-2000-E fluorescence microscope equipped with a Retiga6 camera (Photometrics.com) controlled by Oculus software. Pictures of fluorescent colonies were taken from randomly selected fields and the relative size of the colonies ascertained using Fiji image analysis software (https://ImageJ.github.io) and analyzed with the prism software package. Confocal images were captured using a Zeiss LSM 710 microscope with a 20X objective, Zeiss Zen capture software and Imaris image analysis software (Oxford Instruments, Bitplane).

### Western blotting

SDS-lysed keratinocyte cell lysates were equalized for protein concentration using BioRad reagents. Equal amounts of protein-normalized samples were loaded onto SDS-acrylamide gels, electrophoresed, and transferred onto PVDF membranes. Blots were blocked in 0.05% tween-20/5% Non-fat milk in Tris-buffered saline and probed with the indicated antibodies to GAPDH (#368 from Cell Signaling), Tubulin (#T9026 from BD Biosciences), anti-16E7 (a mix of both monoclonal antibody clones 8C9 and EDV7 from Santa Cruz Biotechnology), and antibodies from ThermoFischer to actin (MS-1295-P1) and p53 (MA1-19055). Anti-AU1 tag monoclonal antibody was a gift of Richard Schlegel (Georgetown University). Anti-16E6 MAb 6G6 was a kind gift of Johannes Schweitzer (Arbor Vita Corp.); lysates for 16E6 western blots were prepared from Igepal-lysed cells as described [45].

## Results

### HPV16 confers enhanced cell competition to keratinocytes

**Fig 1:**
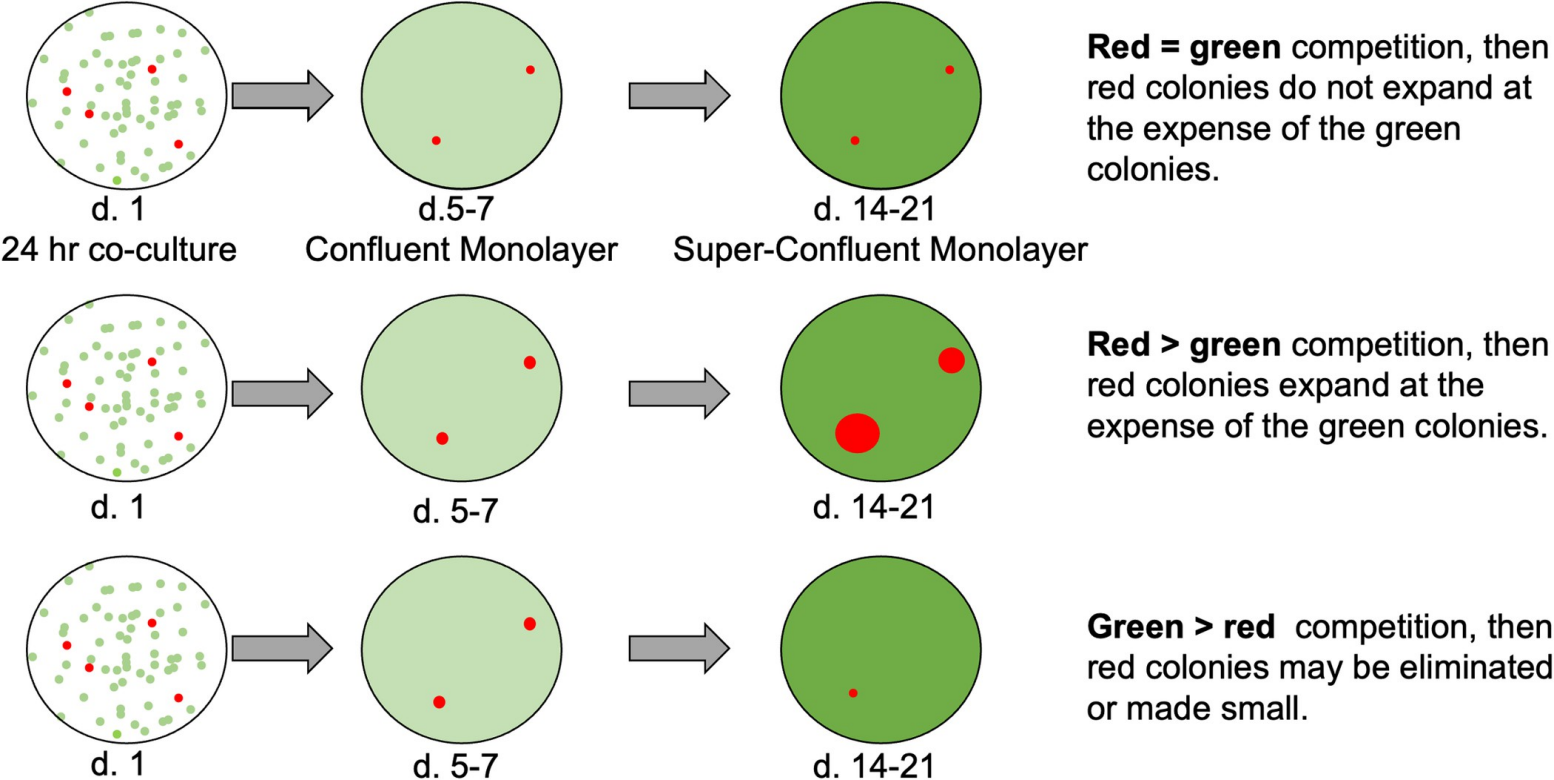
An assay for enhanced competition by papillomavirus infected cells. Keratinocytes were labeled by lentiviral transduction with either green (eGFP) or red (Fusion Red) proteins and then transduced with either vector or papillomavirus expressing retroviruses and cultured separately. On day 1 of the assay, 99.9% vector-expressing green cells and 0.1% oncogene expressing red cells are mixed and plated together at 10% confluency in a 10 cm dish. 24 hours later, those co-plated cells are trypsinized and each sample was plated onto 3 glass coverslips in a 6 well plate (2.1 10^4^ cells / cm^2^). Cells reach confluency by day 5–7 at which point one well is fixed and a second well is fed with F-media for another 7 days and then fixed, and a third well incubated for another 7 days before fixation and staining with DAPI. Pictures of fluorescent colonies are taken with a 4X objective from randomly selected fields and the relative size of the colonies ascertained using NIH ImageJ software.

An assay was developed to mimic the early stage of an HPV16 infection where an infected keratinocyte establishes a nascent papilloma and is illustrated in Fig 1.

0.1% HPV16 transfected and fluorescently-tagged keratinocytes are seeded together with 99.9% vector-transduced keratinocytes fluorescently tagged with a contrasting-colored protein for 24 hrs. before re-trypsinization and re-seeding of the mixed cell population together onto coverslips in a 6-well plate. The 24-hr. co-culture ensures that both populations begin the competition assay on glass coverslips under identical culture conditions. Confluency is reached about 5–7 days after seeding onto coverslips, at which time the first coverslip is fixed and dapi stained. The remaining coverslips are cultured for a further 7–14 days (becoming super-confluent), with the second coverslip fixed on day 14 and the last coverslip fixed on day 21. Over 21 days, the culture stratifies to a 2–4 cell thick epithelium (S1 Fig). A variety of fluorescent proteins were screened for this assay (EGFP, Fusion Red, mCherry, mCitrine, mVenus, and mCerulean) with EGFP and Fusion Red being chosen both for similar toxicity and for spectral properties. The assay duration is limited by eventual detachment of portions of the epithelial sheet from the coverslip after day 21. Pictures were taken of random fields and the relative size of fluorescent colonies calculated and compared to vector-transduced colonies. As super-confluency is reached, the 3T3 feeder cells in the culture are forced off the plate by the keratinocytes and auto-fluoresce in both red and green channels (see below); these balls of auto-fluorescent cells were excluded from analysis.

**Fig 2:**
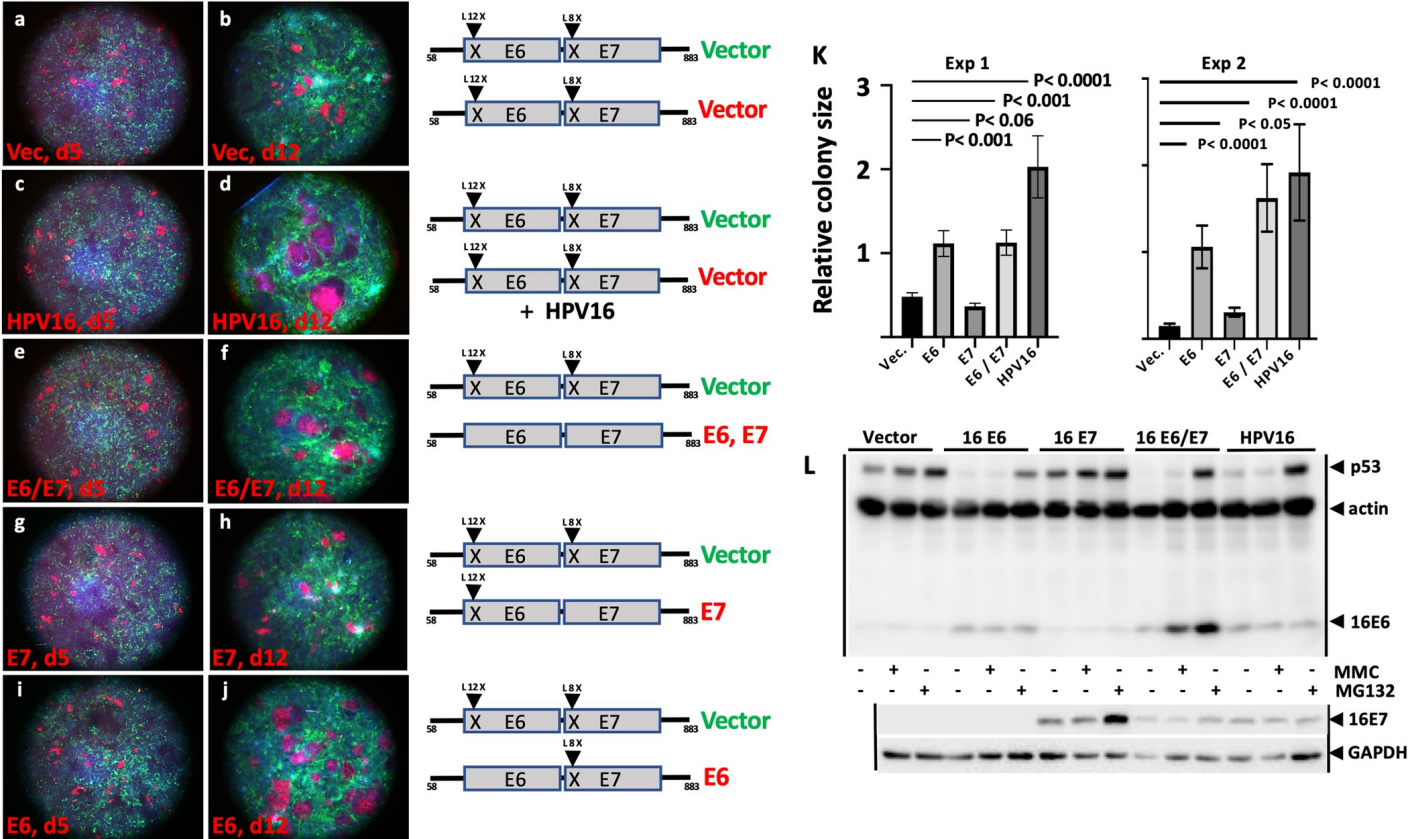
Enhanced cell colony competition is induced by HPV16 and by HPV16 E6. Vector-transduced green NIKS cells and oncogene-expressing red NIKS cells were seeded together as described in Fig 1 onto coverslips on day 2 and fixed at confluency on day 5 (Fig 2A, 2C, 2E, 2G, and 2I); a second coverslip was fixed on day 12 (Fig 2B, 2D, 2F, 2H, and 2J). The transduced, selected and stably expressed genes are indicated to the right of each pair. The quantified results of colony sizes (in arbitrary units) from 2 individual representative experiments (out of a total of 4 experiments) is shown (Fig 2K). Western blots for the stably transduced cell lines selected in Fusion Red expressing cells is shown (Fig 2L). MMC refers to treatment with mitomycin C that induces p53 expression, and MG132 refers to the presence of proteasome inhibitor MG132 which blocks the proteasomal degradation of p53. On Day 5 colony sizes were not statistically different between the samples and are not shown. HPV16 and E6 confer enhanced cell colony size at day 12 while E7 does not. Error bars in Fig 2K depict standard error of the mean for colony sizes.

If HPV16 conferred no competitive advantage, the mean colony area size should not exceed those of vector transduced cells (Fig 1), but that was not the case (Fig 2). HPV16-expressing red keratinocyte colonies expanded in surface area at the expense of already-confluent vector-transduced green cells, while red vector-transduced cells only modestly out-competed green cells (Fig 2C, 2D, and 2K).

### E6 and E7 proteins phenocopy enhanced competition caused by the complete HPV16 genome

HPV16 encodes E5, E6, and E7 oncoproteins as well as RNA products that encode additional proteins and which could have additional non-proteinaceous functions. In most cervical cancers, both the E6 and E7 genes are expressed after viral integration within the E2 or E1 ORFs into the host cell chromosome [46,47]. In order to determine if only the E6 and E7 proteins are sufficient to confer enhanced cell competition, a retrovirus expressing the HPV16 E6 and E7 proteins was introduced into red keratinocytes while an identical retrovirus with stop codons introduced early in the E6 and E7 ORFs was introduced into the green cells in order to insure that the red and green cells express common RNA products and drug selection markers, and differ only in the expression of E6 and E7 proteins and fluorescent markers. Fig 2E, 2F, and 2K show that E6 together with E7 proteins alone conferred enhanced cell competition. Thus, neither E5 (which is not encoded within the HPV16 fragment) nor other virally encoded functions in HPV16 are essential for enhanced cell competition conferred by the HPV16 genome.

### 16E6 predominantly confers enhanced cell competition compared to E7

The indicated retroviral constructions in Fig 2 were used to express only E6 or only E7 in red cells compared to surrounding green cells. While 16E6-expressing red colonies expanded at the expense of green vector-transduced keratinocytes, 16E7-expressing keratinocyte colonies were similar or smaller than vector-transduced keratinocytes. The absence of colony expansion conferred by 16E7 was not due to an absence of E7 expression as both oncoproteins were expressed where expected and not where mutated (Fig 2L). The differences in cell colony sizes were quantified by automated quantification of randomly selected microscopic fields and reached high statistical significance (Fig 2K). Western blots demonstrated that expression levels of 16E6 from retroviruses was similar to expression levels from the episomal HPV16 genome (Fig 2L).

To confirm that the results of Fig 2 were not the result of different fluorescent protein tags, the Fig 2 assay was repeated with the oncoproteins expressed in green cells and the surrounding competing cells tagged with Fusion Red. S2 Fig shows similar competition results regardless of green or red fluorescent tags employed, and S3 Fig shows that untagged keratinocytes compete to a similar degree as the fluorescently tagged keratinocytes shown in Fig 2. The preceding figures show that E6 expressing keratinocyte colonies expand at the expense of already-confluent surrounding cells. S4 Fig shows that the converse is also true, that E6 expressing surrounding cells restrict the expansion of E6 or HPV16 expressing colonies compared to vector-transduced keratinocytes; thus, colony size is dependent upon the traits of both the oncogene-expressing colony and the surrounding cells.

### 16E6 alone confers enhanced competition

**Fig 3:**
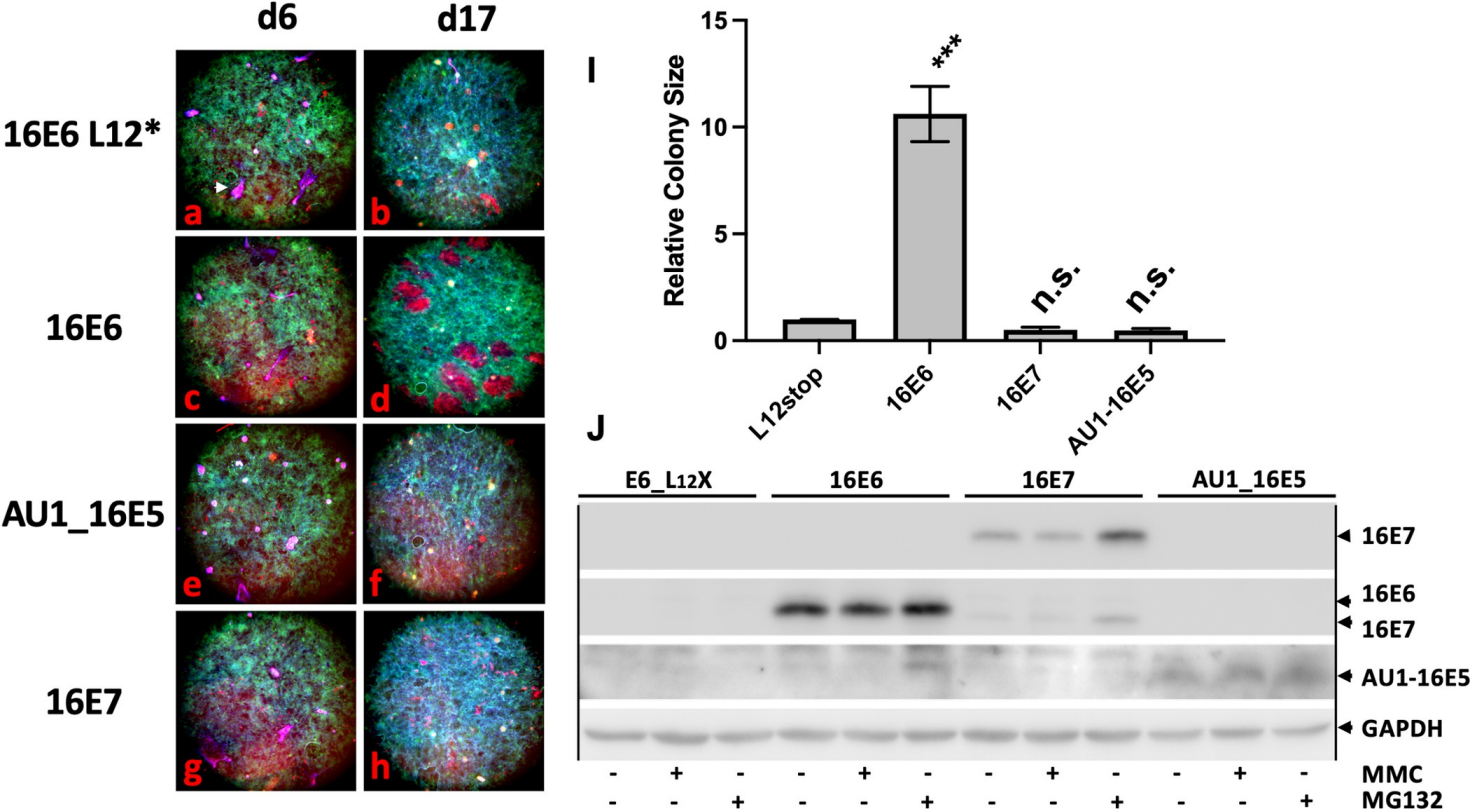
16E6 induces cell colony competition while 16E5 and 16E7 do not. The individual HPV16 oncoproteins were transduced into Fusion-Red tagged NIKS cells and set into competition against EGFP-tagged NIKS cells as described in Fig 1; E5 (Fig 2E and 2F), E6 (Fig 2C and 2D), and E7 (Fig 3G and 3H). 16E6 with a stop codon at amino acid 12 (L12X, Fig 3A and 3B) was the negative vector control. Magenta colonies (indicated with a white arrowhead in Fig 3A) are patches of feeder cells and are not included in the colony analysis. Plates were stained at day 6 when confluent and day 17 when super-confluent. Pictures were taken with a 4X objective and relative colony sizes ascertained at day 17 and shown in arbitrary units (Fig 3I) from 4 separate experiments. Day 6 colony sizes were not statistically different between the samples and are not shown. Error bars represent standard error of the mean. **** is P<0.0001; n.s. is not significant. The pictures shown are representative from one of 4 separate assays. Expression of the papillomavirus oncoproteins in the same cell lines used in this experiment is shown in Fig 3J where the blot was sequentially probed with monoclonal antibodies to E7, E6, and the AU1 epitope on E5 and finally GAPDH.

The absence of the E5 ORF in the retroviral E6/E7 retrovirus used in Fig 2 did not eliminate the possibility that E5 alone might contribute to larger colony sizes. To address this and confirm the validity of the Fig 2 results, red-tagged keratinocytes were transduced with retroviruses expressing only the individual E5, E6, E7 oncogenes or E6 with a stop codon at aa 12 (as the negative control) and set into competition against green keratinocytes. Fig 3 shows that 16E6 alone markedly increased colony sizes while 16E5 or 16E7 did not.

**Fig 4:**
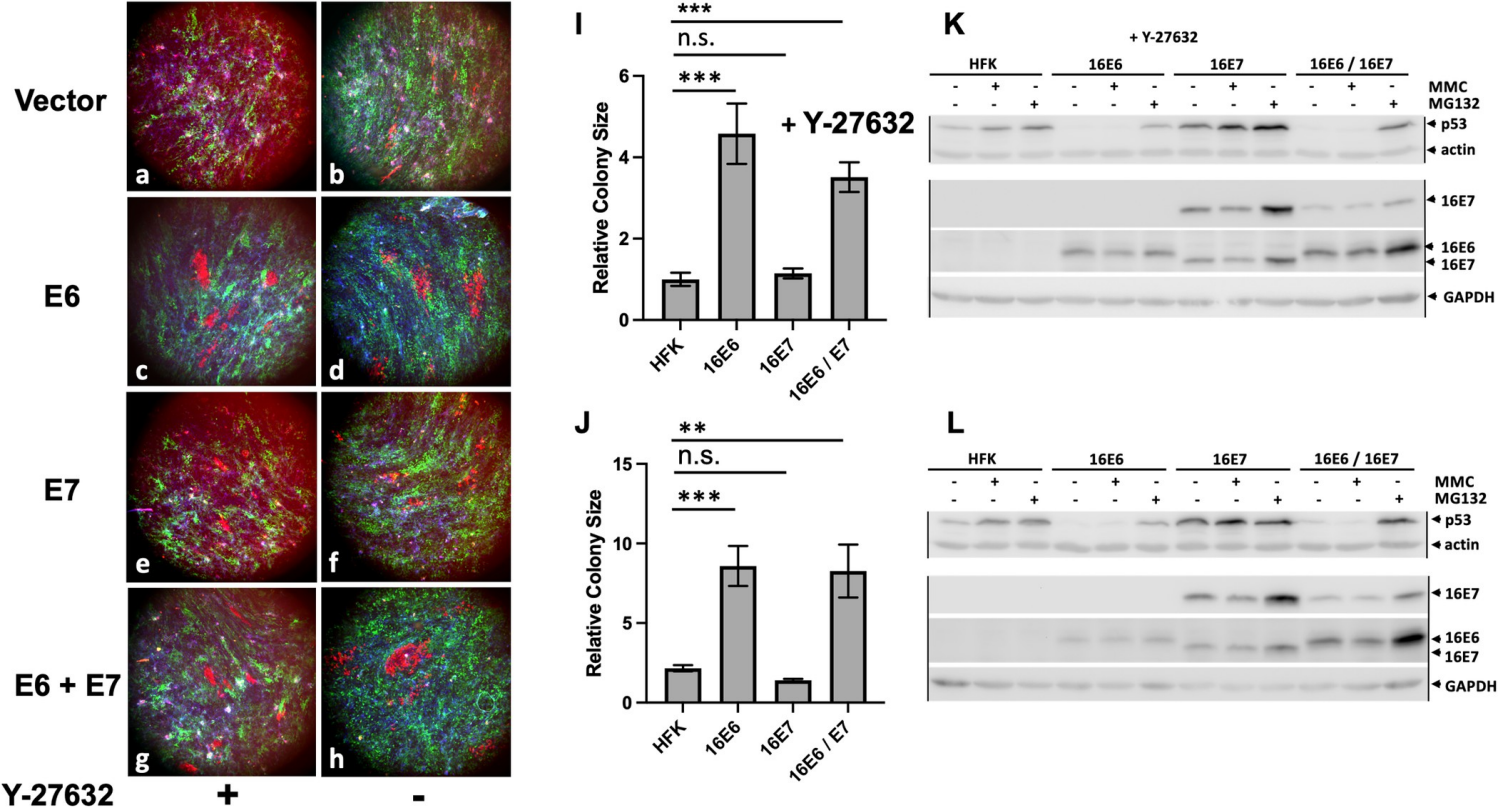
Cell colony competition is induced by E6 in primary keratinocytes. Primary foreskin keratinocytes maintained in the presence of the rho kinase inhibitor Y-27632 were transduced with the indicated fluorescent proteins and oncogenes and seeded onto glass coverslips as described in Fig 1. One set was maintained in media supplemented with Y-27632 (Fig 4A, 4C, 4E, 4G and 4I), but in a duplicate set Y-27632 was removed after seeding onto glass coverslips (Fig 4B, 4D, 4F, 4H, and 4I). Cells were fixed and stained with dapi on day 21 and red colony sizes ascertained by quantitation of photomicrographs in arbitrary units. Error bars represent the variation in colony sizes normalized to vector-transduced cells from 4 experiments and denote standard error of the mean. *** is P<0.001; ** is P<0.0.01; n.s. is not significant. Western blots for expression of p53, actin, 16E6, 16E7, and GAPDH are shown from cells growing in the presence or absence of Y-27632 in parts Fig 4K and 4L respectively.

We wished to ensure that the above results obtained in NIKS keratinocytes were true in primary keratinocytes as well. There is difficulty in transducing primary keratinocytes with non-oncogenic retroviruses as these primary cells senesce after drug selection and several culture passages. To overcome this difficulty, we cultured and transduced primary keratinocytes in F media containing the rho kinase inhibitor Y-27632, which immortalizes primary keratinocytes as long as the drug is continuously present, with the cells resuming a normal cultured-cell limited lifespan when the drug is withdrawn [41]. We transduced and selected primary keratinocytes in the presence of Y-27632, then continued treatment with the drug or removed the drug after the cells were plated onto glass coverslips. Results using primary cells were similar to results using NIKS with the exception that both cell spreading and colony sizes were larger where Y-27632 was removed, and colonies were less cohesive than colonies where Y-27632 was maintained throughout the assay. E6 alone enhanced colony sizes while E7 did not (Fig 4). Western blots confirmed the expected E6 protein expressions (Fig 4K and 4L).

### Mutational analysis of 16E6-induced cell competition

**Fig 5:**
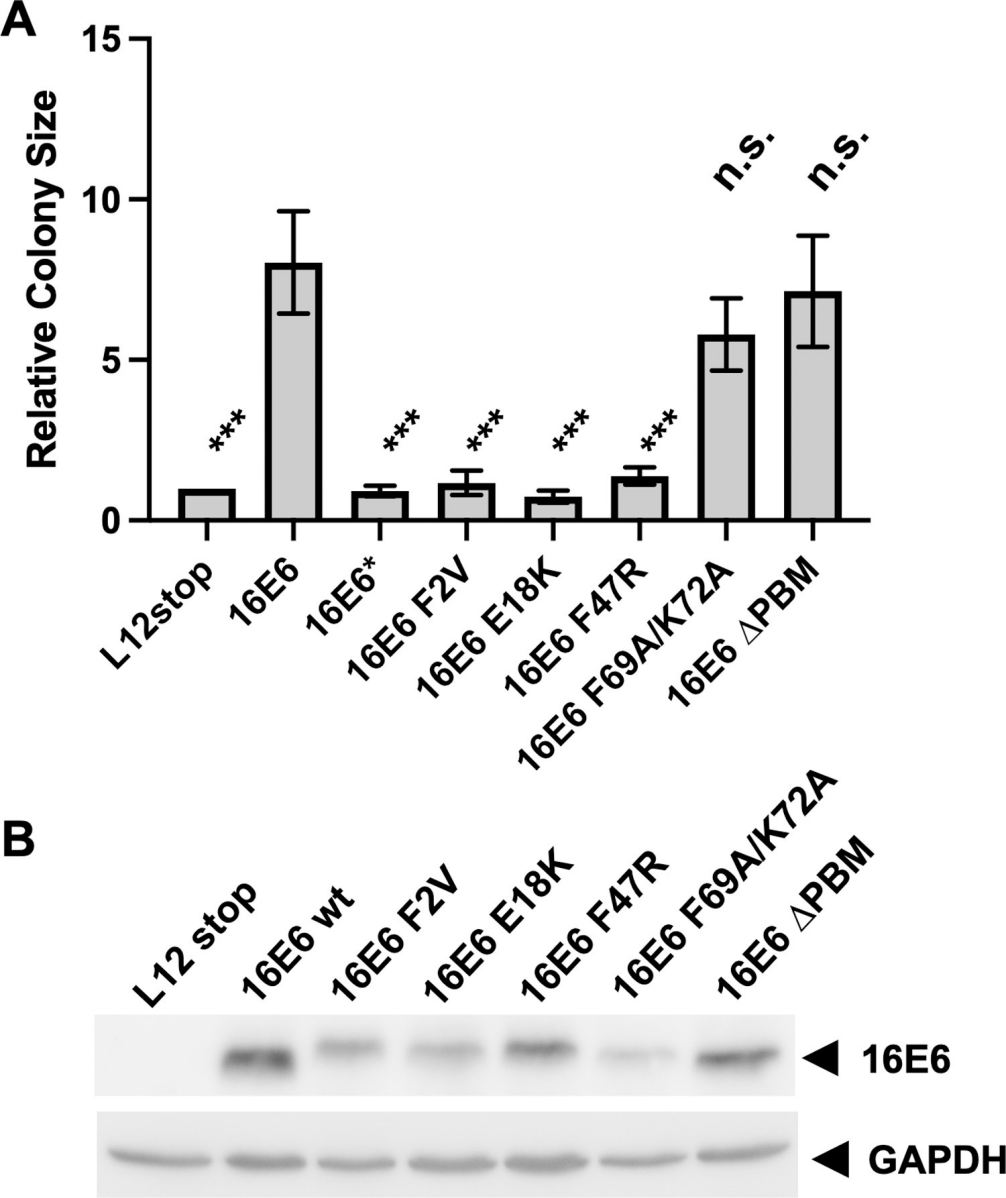
Mutational analysis of 16E6-induced cell colony competition. A. 16E6 mutations that interrupt interaction with p53 (F2V, E18K, F47R), NHERF1 (F69A/K72A), or PDZ-domain proteins (Δ150–151) were introduced into red NIKS keratinocytes and set into competition with green NKS keratinocytes as described in Fig 1. 16E6 with a stop codon at amino acid 12 served as the negative control and colony sizes are normalized to that sample. 16E6 with a stop codon at position 55 (E6*, R55-stop) produces 16E6* but cannot produce full length 16E6. The bar graph combines the results of 4 separate normalized experiments. *** signifies P<0.001, n.s. signifies non-significant differences. Fig 4B. Western blot analysis of 16E6 and mutant 16E6 protein expression. E6* is not shown because it is not recognized by the 6G6 monoclonal anti-16E6 antibody.

16E6 associates with p53, cellular PDZ proteins (including DLG1 and SCRIB), and NHERF1, all of which are implicated in cell competition in other experimental systems [48]. The 16E6 amino acids that interact with these targets have been mapped to the molecular structure of 16E6, can be selectively mutated [49,50], and have been characterized with respect to protein target degradation [49–51]. Another possibility was that a smaller E6 protein termed E6* (that originates from a spliced E6 mRNA) could play a role in colony size. 16E6 mutations that selectively target p53 interaction (F2V, E18K, or F47R), NHERF1 interaction (F69A/K72A), PDZ proteins (Δ150–151), and that truncate full length 16E6 while retaining the E6* form (R55 stop) were introduced into red keratinocytes and set into competition with vector-transduced green keratinocytes. E6* did not enhance colony sizes, while separate mutation of each of the three p53 interaction sites ablated 16E6-induced enhanced colony size. Mutation of neither the NHERF1 interaction site nor the PDZ ligand site of 16E6 significantly altered cell colony size (Fig 5A). 16E6 mutants in these stably transduced cell lines were detected by western blot (Fig 5B).

### 16E6-expressing keratinocytes undermine and displace normal keratinocytes from the basal layer

**Fig 6:**
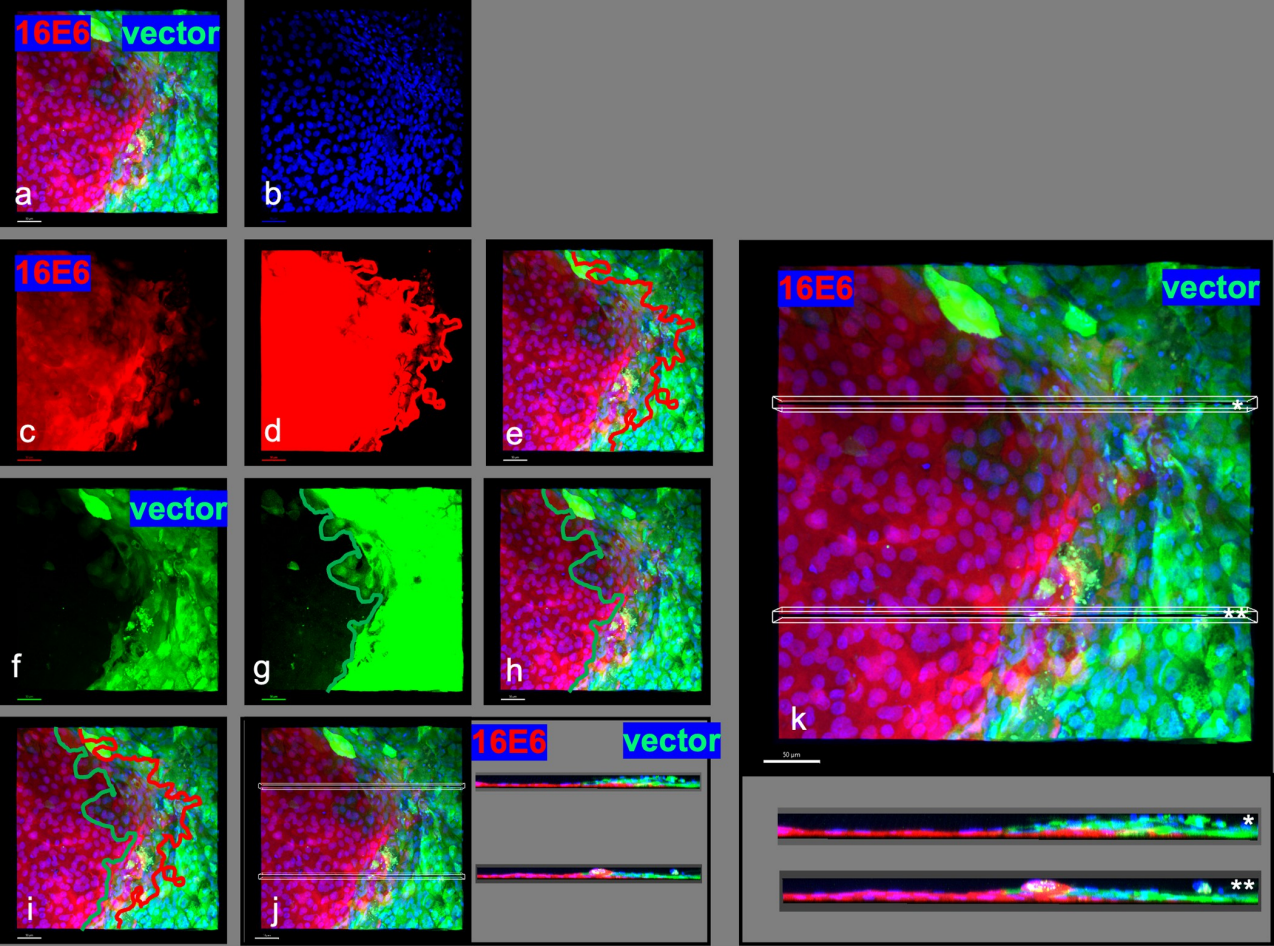
E6-expressing keratinocytes extend basally beneath normal keratinocytes, forcing the normal keratinocytes off the attachment substrate. HPV16 E6 expressing keratinocytes tagged in red were seeded at 0.1% and vector transduced keratinocytes tagged in green were seeded at 99.9% and cultured for 21 days as described in Fig 1. Confocal Images were captured with a 10X objective; the bar at lower left indicates 50 um. Blue color is DAPI stained DNA signal. Fig 6A. Three color image. Fig 6B. DAPI-stained. Fig 6C.! 6E6-transduced cells tagged with Fusion Red. Fig 6D. Overexposure of Fusion Red image with superimposed red line indicating the extent of 16E6 expressing cells. Fig 6E. Red line superimposed onto 3-color image to show the extent of 16E6 expressing cells. Fig 6F. EGFP signal indicating vector-transduced cells. Fig 6G. Overexposure of Fig 6F, with line to indicate extent of vector transduced cells. Fig 6H. Green line from G superimposed onto 3-color image. Fig 6I. 3-color image with red and green lines indicating area of overlapping red-16E6 and green-vector expressing cells. Fig 6J. Three-color image with white boxes indicating the location of z-plane image construction; z-plane images to the right show that 16E6 red cells are basal to vector-green cells. Fig 6K. The same Z-stack image reconstructions shown in Fig 6J are enlarged for clarity; asterix shows the corresponding image and location.

The preceding experiments demonstrate that colonies of 16E6 expressing keratinocytes expand in surface area at the expense of surrounding normal keratinocytes, but how? 16E6- keratinocytes might kill normal keratinocytes, cause normal keratinocytes to retreat en-mass, or displace normal keratinocytes from the basal layer to be displaced upward. To address these possibilities, 16E6- red colonies in competition with vector-green surrounding cells were examined by confocal microscopy. At the margin between the 16E6-red cells and the vector-green cells, areas of overlapping red and green cells were observed (Fig 6D, 6E, 6G, 6H, and 6I). Two separate Z-plane image constructions showed the red E6-cells extended a thin layer basal to the green cells, thereby separating green cells from the coverslip in an apical direction (Fig 6J, duplicated in a larger size for clarity in Fig 6K). A separate colony of 16E6-transduced cells competing against vector-transduced cells shows the same result (S5 Fig) as did cell lines where the fluorescent tags were reversed to ensure that the fluorescent tags employed did not play a role (S6 Fig). All six Z-stack image constructions demonstrated that where the two cell types were in contact, 16E6-transduced cells were extending basally beneath the vector cells, displacing the vector cells apically.

## Discussion

When a papillomavirus infects a basal keratinocyte, the retention of the infected keratinocyte on the basement membrane and expansion of that keratinocyte’s progeny on the basement membrane at the expense of surrounding keratinocytes is a logical prerequisite for the formation of a papilloma. Why papillomaviruses make papillomas at all is curious, and a priori seems unnecessary to produce virus, but a papilloma would both produce more virus-producing cells but also protect virus-producing cells from possibly deleterious interaction with uninfected cells. One can speculate that the interior of a papilloma physically segregates virus-producing cells from contact with uninfected keratinocytes where that interaction might reduce the yield of virus. Our study indicates that expression of E6 confers a competitive advantage for occupation of surface area with E6-expressing cells undermining and displacing vector-transduced cells apically. Loss of substrate attachment results in loss of keratinocyte proliferative capacity and initiates terminal differentiation which is inhibited by integrin signaling [52].

High-risk papillomavirus E6 oncoproteins interact with several mammalian homologs of *Drosophila* proteins that regulate cell competition including p53 [53–56], c-myc [57–61], and cellular PDZ proteins like SCRIB, DLG1, and dPTPMEG (the *Drosophila* homolog of PTPN3) [55,62–66]. Alteration of signaling pathways that influence cell competition in *Drosophila* are also altered by high-risk HPV E6 proteins including WNT [50,67], pI3K/AKT and the Hippo pathway [66,68,69]. Keratinocytes expressing E6 have traits implicated in cell attachment and possibly cell competition; we have previously shown that the HPV16 E6 (16E6) oncoprotein enhances cell attachment and cell spreading by keratinocytes via targeted degradation of p53 [39], which could contribute to enhanced cell colony size competition.

It is surprising that only 16E6 clearly scored in our assay. The assay we developed was designed to illuminate competition for surface area as we hypothesize would be found early in the infectious cycle. The assay is sensitive, and it is important to make sure that the competing cell populations are as nearly equivalent as possible. Other types of cell competition assays (such as those in which cells are admixed and cultured together and/or passaged together) might give rise to differing results than we observed; those assays include additional traits such as rate of cell proliferation and efficiency of cell attachment over multiple tissue culture passages. Different culture conditions might reveal a role for either E5 or E7 that we did not observe. Ablation of 16E6 from the HPV16 genome results in loss of episomal plasmid maintenance, making comparisons in the context of the full HPV16 genome problematic. This assay also provides a means to measure competition between infected and uninfected cells while changing culture conditions or introducing drugs for therapeutic effect.

The lack of clear colony-size competition conferred by E7 in our assay is surprising given that 16E7 scores much more strongly than 16E6 in both anchorage independence of murine 3T3 cells in culture, and E7-induced dysplasia in transgenic mice; it is noteworthy that these are both assays that do not include interaction with normal keratinocytes [27–31].

The lack of cell competition conferred by 16E5 in our assay is also in the context of isolated expression, and results might differ when expressed from the HPV16 genome together with E6. 16E5 overexpression in transgenic murine skin generates differentiation abnormalities that require EGFR signaling [70] and in transgenic mice, E5 potentiates chemical carcinogenesis [71]. Again, it is noteworthy that such transgenic mouse assays exclude interaction of the oncogene-expressing cells with surrounding normal keratinocytes, and there is a natural investigator-bias towards the selection of founder mice with visual abnormalities. It is possible that in cells harboring the episomal HPV16 genome that 16E5 might synergize with E6 to augment competition under as-yet undefined conditions. While neither E5 nor E7 are required for episomal maintenance of HPV16 in keratinocytes [72,73], E6 is required [19,74,75], making a genetic dissection of E6 mutants expressed from the HPV16 genome impossible.

A genetic analysis of retrovirally transduced 16E6 demonstrated that mutation of 16E6 amino acids that directly interact with p53 ablated enhanced colony size (Fig 5). All three separate mutants employed (F2V, E18K and F47R) specifically alter the interaction of 16E6 with p53 without altering the ability of 16E6 to target the degradation of cellular PDZ proteins [49]. Loss of p53 has been implicated in cell competition in the dog kidney MDCK cell line, where winner cells express lower levels of p53, and cell death is mediated in a Rho-kinase dependent manner [55]. In our studies including primary human keratinocytes, 16E6 competed in the absence or presence of the Rho Kinase inhibitor Y-27632 (Fig 4). It is noteworthy that expression of p53 is enhanced by the expression of 16E7 in keratinocytes [76,77]. This may explain both the failure of 16E7-transduced cells to compete with normal keratinocytes and the ability of 16E6 to rescue this trait when co-expressed together with 16E7 (Figs 2 and S2). Our results do not exclude the possibility that 16E6 has additional undefined traits that synergize with p53 degradation to promote cell competition.

While high-risk HPV E6 proteins target the degradation of p53, most genera of papillomaviruses that produce papillomas do not target p53 degradation. Multiple alpha-like genera of E6 proteins do target the degradation of NHERF1 which is a negative regulator of canonical WNT signaling, and enhanced WNT signaling has been shown to enhance cell competition [78]. To our surprise, our assays did not demonstrate a role of NHERF1 or PDZ domain proteins that was independent of p53, even though Hippo signaling and PDZ proteins such as SCRIB and DLG that interact with 16E6 have been implicated in modulating cell competition [64,79]. Most high-risk HPV cervical infections originate within the transition zone between squamous and columnar epithelia, and that 16E6 interactions with NHERF1 and/or PDZ proteins might be important in cell types other than those used in our assays.

In considering papillomavirus types that do not target p53 degradation, there is a split in the papillomavirus evolutionary tree between virus types that encode E6 proteins that associate with the cellular E3 ubiquitin ligase E6AP (such as 16E6 in this study) [80] and papillomavirus types where E6 proteins associate with MAML1 transcriptional co-activators and thereby repress Notch signaling [45,81–83]. E6 proteins that associate with MAML1 may also associate with paxillin, a cellular adapter protein that regulates cell attachment via integrin signaling [84–87]; the expression of paxillin is required for transformation by the BPV-1 E6 protein that interacts with both paxillin and MAML1 [88]. Since Notch signaling controls squamous cell differentiation and carcinogenesis [89] and MAML1 is the transcriptional effector of Notch, the association of many E6 proteins with MAML1 could be a candidate interaction for influencing cell competition; consistent with that model, the targeted expression of a dominant negative MAML1 in the basal squamous epithelium of murine esophagus induces cell competition [90]. Also, the loss of p53 in keratinocytes results in a progressive decrease of Notch1 expression in keratinocytes passaged over time [91,92] as does prolonged expression of HPV16 E6 with consequent loss of p53 protein [92,93], which could play a role in the traits described in the current study. Mutational inactivation of NOTCH1 is the most frequent event in clonal expansion of sun-exposed but histologically normal skin and esophagus [94,95]. By analogy, this suggests that HPV E6 proteins that interact with MAML1 from beta and gamma genera might influence cell competition through repression of notch signaling [81,82]. Indeed, while this manuscript was under review, a study of MmuPV1 demonstrated that the E6 protein of that virus induced cell competition in vivo and in vitro [96]. This suggests the possibility that enhanced cell competition could be a broadly manifested property of many E6 proteins. In the case of the alpha genera human papillomavirus low risk E6 proteins such as HPV11, it is unclear if E6 is involved since those proteins have not been shown to associate with p53, and the E5 oncoproteins of the closely related HPV6 virus have been shown to have oncogenic activity in anchorage independence assays [97]; further experimentation will be required to resolve the genes that regulate competition in low risk HPV types.

### Limitations of this study

There is no perfect model for HPV infected cell competition because we cannot infect people and do genetic analysis of cell competition, so we used an in vitro modeling of cell competition. Human papillomaviruses such as HPV16 give rise to infected cells that outcompete surrounding uninfected cells, and our experiments model this in vitro. Our model allowed a genetic dissection that maps HPV16 induced cell competition to E6 association with p53. Although there are differences in the culture conditions of our assays compared to in vivo, those differences are applicable to both infected and uninfected cells since the outcome of the assay involves both cell types simultaneously. Another important difference between in vitro and in vivo analysis is the attachment substrate to coverslips that are stiff compared to dermis. Animal in vivo models of high-risk HPV have difficulties where animals manifest a trait in all the cells of the animal without competition, and where expression of an oncogene or mutant in different founder animals can be quite different.

A detailed genetic analysis of diverse papillomavirus oncoproteins in cell competition assays may offer new insights into the mechanisms by which squamous cell competition can be regulated.

## Supporting information

S1 Fig NIKS cells grown at prolonged super confluence stratify. NIKS cells grown 14 days past confluency were fixed in formalin and then permeabilized by either 0.1% Tween in PBS (A) or 1% Igepal in PBS (B). Cells were then stained with Alexa-488 labeled phalloidin (green), Alexa-568 E-cadherin (red), and DAPI (blue). In tween-permeabilized cultures, only DAPI penetrates all cell layers, Alexa-488-phalloidin primarily stans the top layer of stratified squamous cells, and there is minimal penetration phalloidin or of Alexa-568 labeled antibody to E-cadherin to the basal layer. In Igepal-permeabilized cultures, all three stains labelled full thickness.
(DOCX)

S2 Fig Cell competition induced by HPV16 or HPV16 E6 alone is similar in competing cells expressing either EGFP or Fusion Red tags. The same oncogene transductions shown in Fig 2 were expressed in either Fusion Red (S2A Fig) or EGFP tagged cells (S2B Fig) and put into competition with the alternate-colored cells as shown and described in Fig 2. Colony sizes are shown in arbitrary units and error is standard error of the mean. **** is P<0.0001; n.s. is not significant.
(DOCX)

S3 Fig Fluorescent tagging of competing keratinocytes does not prevent cell competition by 16E6 expressing keratinocytes. EGFP-tagged NIKS cells expressing either 16E6 or the complete episomal HPV16 genome compete efficiently against un-tagged NIKS cells. Relative colony sizes on day 17 are shown in arbitrary units (S3G Fig) and error bars represent standard error of the mean. **** is P<0.0001; n.s. is not significant.
(DOCX)

S4 Fig Expression of HPV16 E6 in surrounding cells restricts colony formation. Fusion-red-tagged colony-forming cells were seeded at 0.1% of cell number compared to the indicated 99.9% EGFP-expressing surrounding cells as described in Fig 1 and cultured for 21 days prior to fixation. Green surrounding cells express either vector, 16E6, or the complete HPV16 genome as indicated. Expression of either HPV16 or 16E6 restricts colony formation compared to surrounding vector cells. Results shown are the normalized average of 2 experiments with the error bars indicating the range of the results.
(DOCX)

S5 Fig E6-expressing keratinocytes extend basally beneath normal keratinocytes, forcing the normal keratinocytes off the attachment substrate. HPV16 E6 expressing keratinocytes tagged in red were seeded at 0.1% and vector transduced keratinocytes tagged in green were seeded at 99.9% and cultured for 21 days as described in Fig 1. Confocal Images were captured with a 10X objective; the bar at lower left indicates 50 um. Blue color is DAPI stained DNA signal. The three-color image with white boxes indicates the location of z-plane image construction; adjacent z-plane images show that 16E6 red cells are basal to vector-green cells.
(DOCX)

S6 Fig E6-expressing EGFP-labeled -keratinocytes extend basally beneath normal-red-tagged keratinocytes, forcing the normal keratinocytes off the attachment substrate. HPV16 E6 expressing keratinocytes tagged in green were seeded at 0.1% and vector transduced keratinocytes tagged in red were seeded at 99.9% and cultured for 21 days as described in Fig 1. Confocal Images were captured with a 10X objective; the bar at lower left indicates 50 um. Blue color is DAPI stained DNA signal. The three-color image with white boxes indicates the location of z-plane image construction; adjacent z-plane images show that 16E6 green cells are basal to vector-red cells.
(DOCX)
