# Supplementary material for: Human papillomavirus type 16 E6 induces cell competition
Source: PLoS Pathog. 2022 Mar 23;18(3):e1010431. doi: 10.1371/journal.ppat.1010431 (PMC8979454; doi:10.1371/journal.ppat.1010431)
Supplement: S1 Fig — NIKS cells grown 14 days past confluency were fixed in formalin and then permeabilized by either 0.1% Tween in PBS (A) or 1% Igepal in PBS (B). Cells were then stained with Alexa-488 labeled phalloidin (green), Alexa-568 E-cadherin (red), and DAPI (blue). In tween-permeabilized cultures, only DAPI penetrates all cell layers, Alexa-488-phalloidin primarily stans the top layer of stratified squamous cells, and there is minimal penetration phalloidin or of Alexa-568 labeled antibody to E-cadherin to the basal layer. In Igepal-permeabilized cultures, all three stains labelled full thickness. (DOCX) [file ppat.1010431.s001.docx]

**S1 Fig. NIKS cells grown at prolonged super confluence stratify.** NIKS cells grown 14 days past confluency were fixed in formalin and then permeabilized by either 0.1% Tween in PBS (A) or 1% Igepal in PBS (B). Cells were then stained with Alexa-488 labeled phalloidin (green), Alexa-568 E-cadherin (red), and DAPI (blue). In tween-permeabilized cultures, only DAPI penetrates all cell layers, Alexa-488-phalloidin primarily stans the top layer of stratified squamous cells, and there is minimal penetration phalloidin or of Alexa-568 labeled antibody to E-cadherin to the basal layer. In Igepal-permeabilized cultures, all three stains labelled full thickness.
