# Supplementary material for: Human papillomavirus type 16 E6 induces cell competition
Source: PLoS Pathog. 2022 Mar 23;18(3):e1010431. doi: 10.1371/journal.ppat.1010431 (PMC8979454; doi:10.1371/journal.ppat.1010431)
Supplement: S2 Fig — The same oncogene transductions shown in Fig 2 were expressed in either Fusion Red (S2A Fig) or EGFP tagged cells (S2B Fig) and put into competition with the alternate-colored cells as shown and described in Fig 2. Colony sizes are shown in arbitrary units and error is standard error of the mean. **** is P<0.0001; n.s. is not significant. (DOCX) [file ppat.1010431.s002.docx]

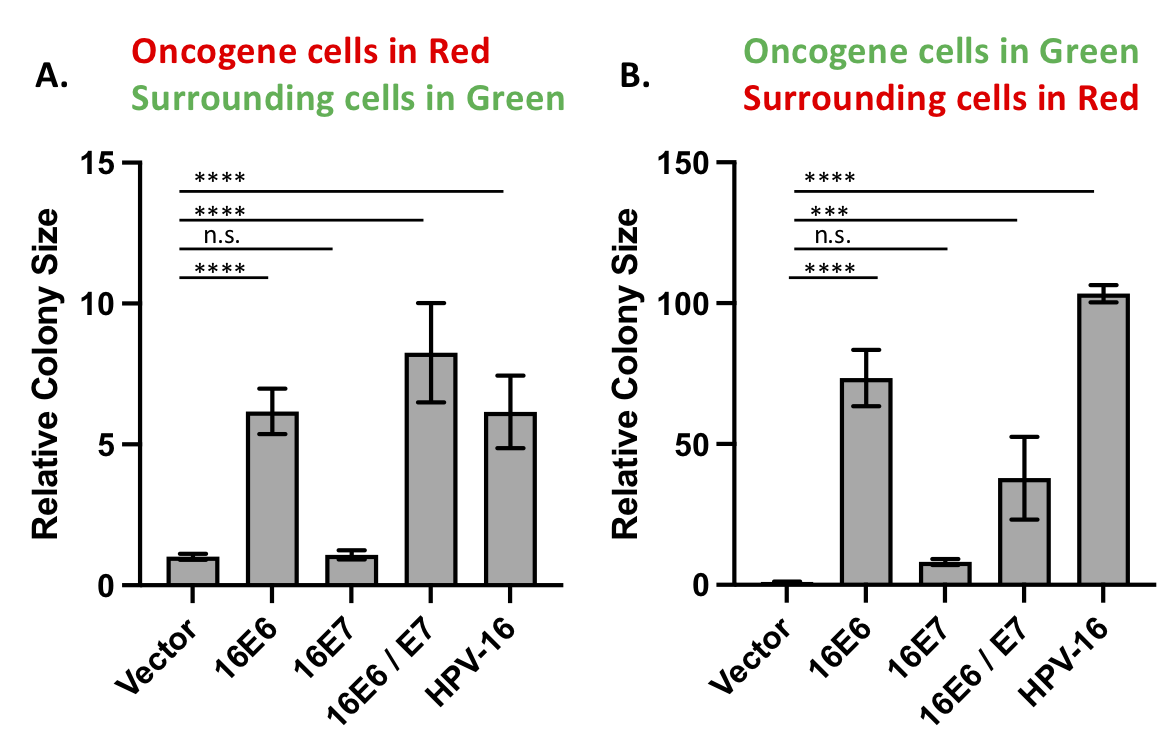


**S2 Fig. Cell competition induced by HPV16 or HPV16 E6 alone is similar in competing cells expressing either EGFP or Fusion Red tags.** The same oncogene transductions shown in Fig. 2 were expressed in either Fusion Red (S2A Fig) or EGFP tagged cells (S2B Fig) and put into competition with the alternate-colored cells as shown and described in Fig. 2. Colony sizes are shown in arbitrary units and error is standard error of the mean. **** is P<0.0001; n.s. is not significant.
