# Supplementary material for: Human papillomavirus type 16 E6 induces cell competition
Source: PLoS Pathog. 2022 Mar 23;18(3):e1010431. doi: 10.1371/journal.ppat.1010431 (PMC8979454; doi:10.1371/journal.ppat.1010431)
Supplement: S3 Fig — EGFP-tagged NIKS cells expressing either 16E6 or the complete episomal HPV16 genome compete efficiently against un-tagged NIKS cells. Relative colony sizes on day 17 are shown in arbitrary units (S3G Fig) and error bars represent standard error of the mean. **** is P<0.0001; n.s. is not significant. (DOCX) [file ppat.1010431.s003.docx]

**
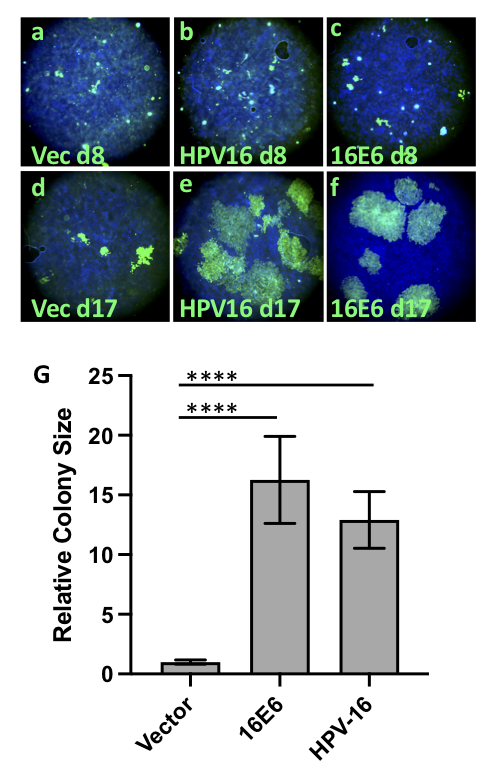
**

**S3 Fig. Fluorescent tagging of competing keratinocytes does not prevent cell competition by 16E6 expressing keratinocytes.** EGFP-tagged NIKS cells expressing either 16E6 or the complete episomal HPV16 genome compete efficiently against un-tagged NIKS cells. Relative colony sizes on day 17 are shown in arbitrary units (S3G) and error bars represent standard error of the mean. **** is P<0.0001; n.s. is not significant.
