# Supplementary material for: Human papillomavirus type 16 E6 induces cell competition
Source: PLoS Pathog. 2022 Mar 23;18(3):e1010431. doi: 10.1371/journal.ppat.1010431 (PMC8979454; doi:10.1371/journal.ppat.1010431)
Supplement: S4 Fig — Fusion-red-tagged colony-forming cells were seeded at 0.1% of cell number compared to the indicated 99.9% EGFP-expressing surrounding cells as described in Fig 1 and cultured for 21 days prior to fixation. Green surrounding cells express either vector, 16E6, or the complete HPV16 genome as indicated. Expression of either HPV16 or 16E6 restricts colony formation compared to surrounding vector cells. Results shown are the normalized average of 2 experiments with the error bars indicating the range of the results. (DOCX) [file ppat.1010431.s004.docx]

**S4 Fig. Expression of HPV16 E6 in surrounding cells restricts colony formation.** Fusion-red-tagged colony-forming cells were seeded at 0.1% of cell number compared to the indicated 99.9% EGFP-expressing surrounding cells as described in Fig 1 and cultured for 21 days prior to fixation. Green surrounding cells express either vector, 16E6, or the complete HPV16 genome as indicated. Expression of either HPV16 or 16E6 restricts colony formation compared to surrounding vector cells. Results shown are the normalized average of 2 experiments with the error bars indicating the range of the results.
