# Supplementary material for: Human papillomavirus type 16 E6 induces cell competition
Source: PLoS Pathog. 2022 Mar 23;18(3):e1010431. doi: 10.1371/journal.ppat.1010431 (PMC8979454; doi:10.1371/journal.ppat.1010431)
Supplement: S6 Fig — HPV16 E6 expressing keratinocytes tagged in green were seeded at 0.1% and vector transduced keratinocytes tagged in red were seeded at 99.9% and cultured for 21 days as described in Fig 1. Confocal Images were captured with a 10X objective; the bar at lower left indicates 50 um. Blue color is DAPI stained DNA signal. The three-color image with white boxes indicates the location of z-plane image construction; adjacent z-plane images show that 16E6 green cells are basal to vector-red cells. (DOCX) [file ppat.1010431.s006.docx]

**S6 Fig. E6-expressing EGFP-labeled -keratinocytes extend basally beneath normal-red-tagged keratinocytes, forcing the normal keratinocytes off the attachment substrate.** HPV16 E6 expressing keratinocytes tagged in green were seeded at 0.1% and vector transduced keratinocytes tagged in red were seeded at 99.9% and cultured for 21 days as described in Fig 1. Confocal Images were captured with a 10X objective; the bar at lower left indicates 50 um. Blue color is DAPI stained DNA signal. The three-color image with white boxes indicates the location of z-plane image construction; adjacent z-plane images show that 16E6 green cells are basal to vector-red cells.
